# Thoracic posture-related morphological changes in patients with pectus excavatum versus healthy controls

**DOI:** 10.1093/ejcts/ezae408

**Published:** 2024-11-12

**Authors:** Takahiro Suzuki, Keisuke Asakura, Yoshitake Yamada, Kyohei Masai, Minoru Yamada, Yoichi Yokoyama, Yu Okubo, Kaoru Kaseda, Tomoyuki Hishida, Masahiro Jinzaki

**Affiliations:** Division of Thoracic Surgery, Department of Surgery, Keio University School of Medicine, Tokyo, Japan; Division of Thoracic Surgery, Department of Surgery, Keio University School of Medicine, Tokyo, Japan; Department of Radiology, Keio University School of Medicine, Tokyo, Japan; Division of Thoracic Surgery, Department of Surgery, Keio University School of Medicine, Tokyo, Japan; Department of Radiology, Keio University School of Medicine, Tokyo, Japan; Department of Radiology, Keio University School of Medicine, Tokyo, Japan; Division of Thoracic Surgery, Department of Surgery, Keio University School of Medicine, Tokyo, Japan; Division of Thoracic Surgery, Department of Surgery, Keio University School of Medicine, Tokyo, Japan; Division of Thoracic Surgery, Department of Surgery, Keio University School of Medicine, Tokyo, Japan; Department of Radiology, Keio University School of Medicine, Tokyo, Japan

**Keywords:** Pectus excavatum, Upright CT, Postural changes

## Abstract

**OBJECTIVES:**

Cases of severe pectus excavatum presenting with worsening cardiopulmonary symptoms in the upright position have been reported. However, the underlying mechanism remains unclear. We evaluated posture-related morphological changes of the thorax in patients with pectus excavatum.

**METHODS:**

Chest morphology was evaluated preoperatively using upright and supine computed tomography in 21 patients with pectus excavatum and 35 healthy volunteers. The minimum anterior–posterior thoracic diameter, depression depth, and Haller index on horizontal sections, as well as the T6-12 plumb line distance on sagittal sections, were compared between the 2 positions.

**RESULTS:**

In patients with pectus excavatum (median age, 22 years; 18 males and 3 females), the minimum anterior–posterior diameter was smaller (4.5 vs 5.1 cm, *P* < 0.001) and the Haller index was greater (10.1 vs 6.4, *P* < 0.001) in the upright position than in the supine position. The T6-T12 plumb line distance was longer in the upright position (2.4 vs 0.8 cm, *P* < 0.001), while the depression depth showed no significant difference. Healthy volunteers exhibited similar changes. The degree of spinal curvature increased in the upright position due to the anterior shift of the lower thoracic vertebrae, resulting in a shorter anterior–posterior diameter, irrespective of chest wall deformity. One patient with dyspnoea only in the upright position exhibited compression of the right inferior pulmonary vein only on upright computed tomography.

**CONCLUSIONS:**

The minimum anterior–posterior diameter is shorter in the upright position. This may explain the worsening of cardiopulmonary symptoms in patients with severe pectus excavatum when in an upright position.

Clinical trial registration number: UMIN000036438

https://center6.umin.ac.jp/cgi-open-bin/ctr_e/ctr_view.cgi?recptno=R000041519

## Graphical Abstract

**Figure:**
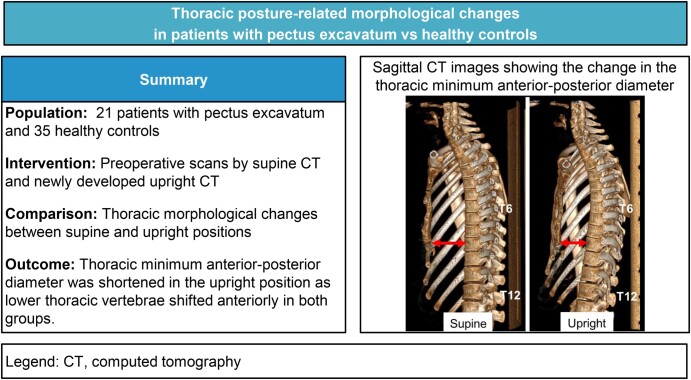

## INTRODUCTION

Pectus excavatum (PE) is a congenital deformity of the anterior chest wall, with a prevalence of at least 1 per 1000 people [1]. Despite most asymptomatic patients expressing dissatisfaction with their appearance [2], some patients with severe chest depression present with dyspnoea, palpitations and chest pain, especially on exertion [3, 4]. The percentage of patients with PE reporting symptoms ranged from 30% to 75% in previous studies [5, 6]. Exercise tolerance is lower in patients with PE than in healthy volunteers [7, 8], and some patients may exhibit improvements following surgery [9].

Postural changes influence circulatory dynamics and may interfere with cardiopulmonary symptoms or exercise capacity in patients with PE. Underwood *et al.* [10] reported a case in which cardiac compression worsened from the supine to the sitting position, resulting in right ventricular outflow tract obstruction. Beiser *et al.* [11] reported decreased cardiac output during upright exercise compared to supine exercise in patients with PE. Therefore, we hypothesize that postural changes can affect cardiovascular compression and symptoms in patients with severe chest depression. However, the underlying mechanism remains unclear.

In conventional supine computed tomography (CT), the Haller index (HI) is widely used to evaluate chest depression severity in cases of PE [12]. However, assessing chest depression morphology in the upright position is impossible with supine CT. We recently developed a CT method to evaluate the anatomical structure of the human skeleton and organs in the upright position, as well as the influence of gravity on the human body [13]. Using this method, we observed posture-related changes in spinal canal stenosis and foramen size in a patient with spondylolisthesis and lumbar foraminal stenosis [13], as well as changes in lung volumes in healthy volunteers and cohorts with chronic obstructive pulmonary disease [14–16]. Therefore, we hypothesized that upright CT would enable evaluation of morphology of the chest wall and organs in patients with PE to ascertain the degree of cardiovascular compression in patients with severe chest depression. In this study, we conducted an exploratory evaluation of posture-related changes in chest depression and organ morphology in patients with PE using upright and supine CT. Additionally, we referred to data from healthy volunteers without PE who underwent upright and supine CT for a better understanding of these morphological changes.

## PATIENTS AND METHODS

### Study population

This prospective single-centre study (UMIN000036438) was approved by the Keio University School of Medicine Ethics Committee, Tokyo, Japan [no. N20160384 (approved on 17 March 2017) and 20180302 (approved on 9 April 2019)]. Written informed consent was obtained from all patients and healthy volunteers. We analysed upright and supine CT data from patients with PE between August 2020 and December 2021 and healthy volunteers without PE between March 2018 and October 2018. All consecutive patients with PE who consented to undergo preoperative CT in both the upright and supine positions were included in this study. The exclusion criteria were as follows: age <16 years, pregnancy, and an inability to undergo CT in the standing position. Regarding healthy control volunteers, we secondarily analysed the data of participants enrolled in a preceding study [15, 17] (no. N20160384) to minimize the number of individuals exposed to radiological procedures. Approximately equal numbers of males and females from the third to sixth decades of life were enrolled. Volunteers with any symptoms, surgical history, or currently undergoing treatment were excluded to ensure the evaluation of normal anatomy. Statistical matching for patient characteristics was not performed owing to the limited number of enrolled volunteers and potential age differences between the 2 inclusion criteria. Data regarding preoperative symptoms was extracted from each patient medical records.

### Physiological function tests

All participants underwent preoperative pulmonary function tests using a spirometer in the sitting position. The presence of restrictive ventilatory impairment (vital capacity <80%) or obstructive ventilatory impairment (forced expiratory volume in the first second <70%) was examined.

### CT examination

**Figure 1:**
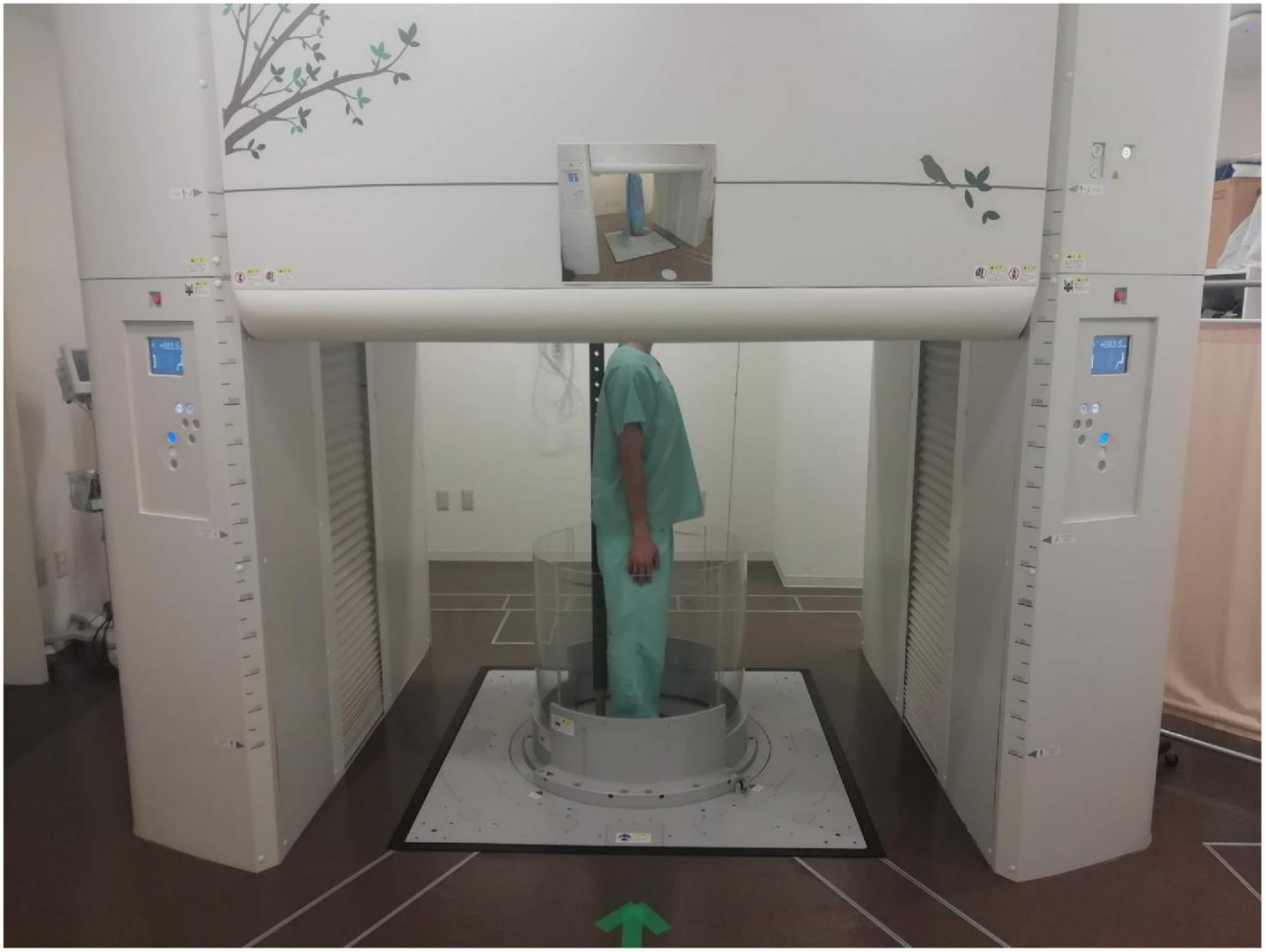
A 320-detector-row upright computed tomography (CT) scanner (prototype TSX-401R; Canon Medical Systems). Upright CT examinations were performed with the patient arms down.

**Figure 2:**
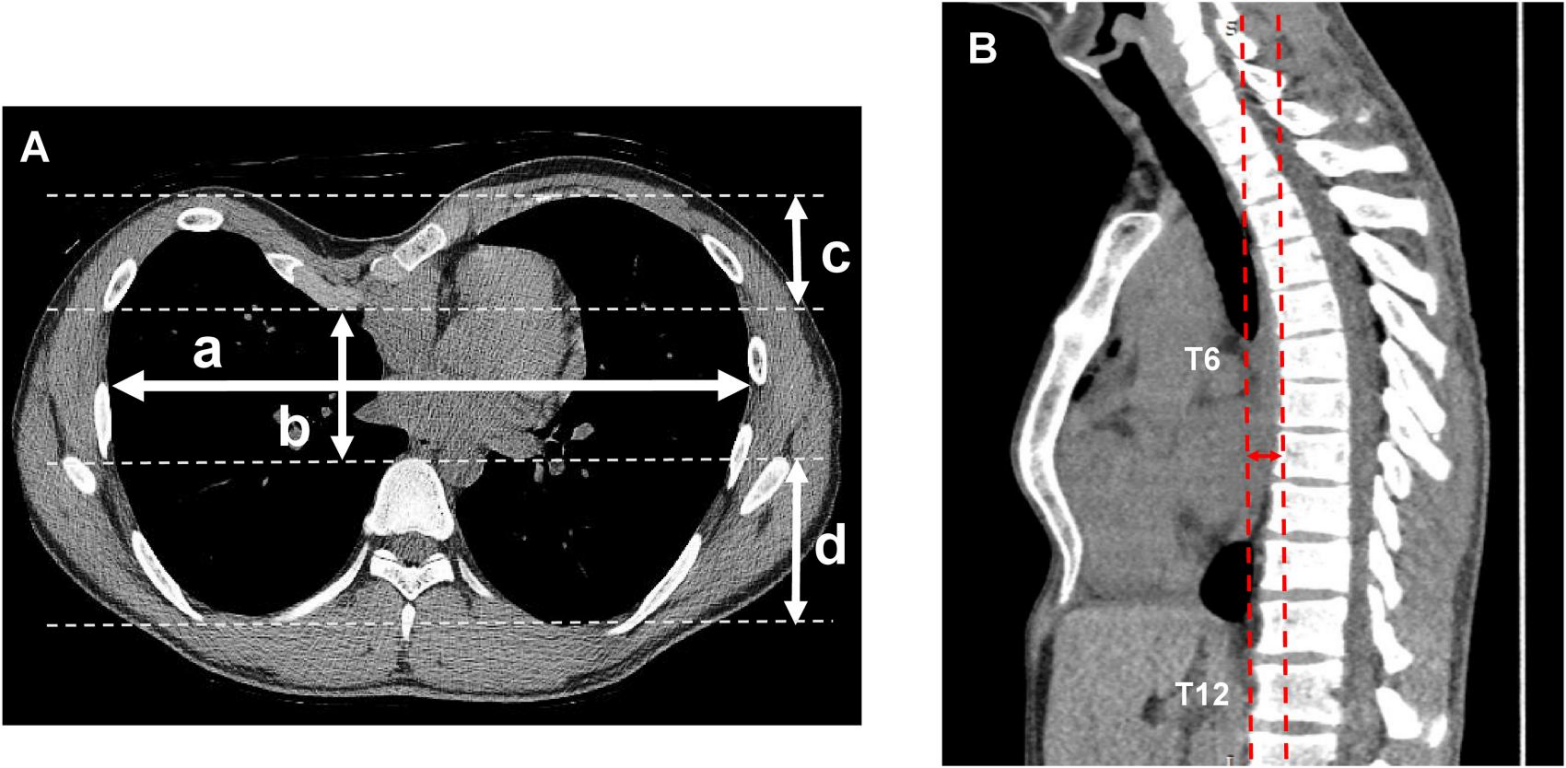
Methods for measuring variables related to pectus excavatum on axial (**A**) and sagittal images (**B**). (**A**) The following parameters were measured on axial images at the level of maximum chest depression: **a**: maximum transverse internal thoracic diameter. **b**: minimum anterior–posterior diameter perpendicular to **a**. **c**: distance between the anterior aspect of the thoracic cavity and the posterior aspect of the sternum. **d**: distance between the anterior aspect of the vertebra and the posterior aspect of the thoracic cavity. (**B**) Sagittal distance between the T6 and T12 plumb lines: The double arrow indicates the distance between the plumb lines along the most anterior points of T6 and T12 in sagittal images at the level of the midline of the spine column.

All participants underwent low-dose chest CT in the supine position with arms down using a 320-detector-row CT scanner (Aquilion ONE, Canon Medical Systems, Otawara, Japan) and low-dose chest CT in the upright position with arms down using a 320-detector-row upright CT scanner (prototype TSX-401R; Canon Medical Systems, Fig. 1) within 1 h on the same day. The upright CT scanner was certified as a medical device in Japan in 2017. As of October 2024, 4 units are in operation in Japan. CT scans were obtained during deep inspiration breath holds. One patient with dyspnoea in the upright position underwent enhanced CT for further evaluation of cardiovascular impairment. All participants were instructed to stand without leaning on the pole behind them and maintain their gaze on a ball placed at eye level during CT scanning to minimize the influence of leaning in the upright position and the shape of the vertebrae on the reliability of measurements. The sagittal images from each case were reconstructed with a slice thickness of 0.5 mm using commercially available software (Ziostation2; Ziosoft, Tokyo, Japan). The following measurements were obtained from axial images at the level exhibiting the maximal value for chest depression (Fig. 2A):

- maximum transverse internal thoracic diameter,
- minimum anterior–posterior diameter perpendicular to *a,*
- distance between the inner margin of the most anterior portion of the chest and the posterior aspect of the sternum and
- distance between the anterior aspect of the vertebra and the inner margin of the most posterior portion of the chest.

The HI is a standard index of PE severity calculated as *a*/*b* [12]. To assess the degree of spinal curvature, we further measured the distance between the T6 and T12 plumb lines (i.e. the distance between the plumb lines along the most anterior points of the T6 and T12 vertebral bodies) at the midline of the spine column on sagittal images (Fig. 2B).

These measurements were performed by 2 thoracic surgeons (T.S. and K.A.) in consensus, under the guidance of radiologists. Although they evaluated the CT images in a randomized manner, blinding of the positions (upright or supine) was difficult due to the presence of a pole for participants in the upright position or a CT scanner table for those in the supine position.

### Statistical analysis

Continuous variables were compared using the Mann–Whitney *U* or Wilcoxon signed-rank sum test, and categorical variables were analysed using the chi-squared test with Yates’ correction when appropriate. *P* < 0.05 was considered statistically significant. All statistical analyses were performed using JMP^®^15 (SAS Institute, Cary, NC).

## RESULTS

### Characteristics of the study participants

Data of 21 patients with PE and 35 healthy volunteers without PE were analysed. Baseline characteristics of the study participants are shown in Table 1. More patients with PE were younger, male, and had a lower body mass index compared to healthy volunteers (*P* < 0.001, *P* = 0.003 and *P* = 0.005, respectively). Eight of 21 (38%) patients with PE were symptomatic, and 1 patient presented with dyspnoea that worsened in the upright position.

**Table 1:** Baseline characteristics of the study participants

Variables	Patients with pectus excavatum (*n* = 21)	Volunteers without pectus excavatum (*n* = 35)	*P*-value
Median age (range), years	22 (16–78)	50 (30–68)	<0.001
Sex, *n* (%)			0.003
Male	18 (86)	16 (46)	
Female	3 (14)	19 (54)	
Body mass index (kg/m^2^), mean±SD	19.5 ± 3.1	22.7 ± 3.1	0.005
Impaired respiratory function, *n* (%)	11 (58)	2 (6)	<0.001
Restrictive ventilatory impairment,^a^*n* (%)	11 (58)	0 (0)	–
Obstructive ventilatory impairment,^b^*n* (%)	1 (5)	2 (6)	
Presence of symptoms, *n* (%)	8 (38)	0 (0)	<0.001
Dyspnoea	4 (19)	0 (0)	
Chest pain	3 (14)	0 (0)	
Palpitation	1 (5)	0 (0)	
Symptoms worsening in the upright position, n (%)	1 (5)	0 (0)	

a Defined as vital capacity <80%.

b Defined as forced expiratory volume in the first second <70%.

Table 2 shows comparisons of anatomical measurements between the patients with PE and healthy volunteers in each position. The HI (Fig. 2A, a and b) was significantly greater, while the minimum anterior–posterior diameter (Fig. 2A and B), an element of the HI, was significantly smaller in the patients than in the healthy volunteers on both upright and supine CT (*P* < 0.001 in all comparisons).

**Table 2:** Comparison of anatomical measurements between patients with pectus excavatum and volunteers

	Upright position			Supine position		
Variables	Patients with pectus excavatum (*n* = 21)	Volunteers (*n* = 35)	*P*-value	Patients with pectus excavatum (*n* = 21)	Volunteers (*n* = 35)	*P*-value
Haller index	10.1 ± 16.7 (3.1 to 80.7)	2.9 ± 0.6 (2.1 to 4.7)	<0.001	6.4 ± 4.4 (2.8 to 22.2)	2.8 ± 0.5 (2.0 to 4.0)	<0.001
Minimum anterior to posterior diameter (cm)	4.5 ± 1.9 (0.3 to 9.1)	8.8 ± 1.9 (5.1 to 12.8)	<0.001	5.1 ± 1.9 (1.1 to 10.1)	9.2 ± 1.9 (5.9 to 13.3)	<0.001
Transverse diameter (cm)	25.9 ± 2.2 (21.1 to 32.0)	24.7 ± 2.0 (21.5 to 29.2)	0.032	26.1 ± 2.2 (21.4 to 31.9)	25.0 ± 2.0 (21.6 to 30.1)	0.074
Vertebrae to thoracic cavity distance (cm)	6.0 ± 0.6 (4.3 to 7.4)	6.5 ± 0.6 (4.8 to 7.3)	0.832	5.6 ± 0.5 (4.4 to 6.7)	5.6 ± 0.5 (4.7 to 6.8)	0.799
Distance between T6 and T12 plumb lines (cm)	2.4 ± 1.0 (0.2 to 4.0)	2.7 ± 1.1 (0.0 to 4.8)	0.330	0.8 ± 0.7 (−0.5 to 2.2)	1.5 ± 1.1 (0.0 to 6.0)	0.010

Values are expressed as mean±SD (range).

### Comparison of anatomical measurements between upright and supine CT

Table 3 presents the results of anatomical measurements between the supine and the upright positions in each group. The HI was greater (mean ± SD: 10.1 ± 16.7 vs 6.4 ± 4.4, *P* < 0.001), and the minimum anterior–posterior diameter was lower in the upright position than in the supine position (4.5 ± 1.9 vs 5.1 ± 1.9 cm, *P* < 0.001). In contrast, no significant differences were observed in the transverse diameter.

**Table 3:** Comparison of anatomical measurements between the upright and supine positions

	Patients with pectus excavatum			Volunteers		
Variables	Upright	Supine	*P*-value	Upright	Supine	*P*-value
Haller index	10.1 ± 16.7 (3.1 to 80.7)	6.4 ± 4.4 (2.8 to 22.2)	<0.001	2.9 ± 0.6 (2.1 to 4.7)	2.8 ± 0.5 (2.0 to 4.0)	0.001
Minimum anterior to posterior diameter (cm)	4.5 ± 1.9 (0.3 to 9.1)	5.1 ± 1.9 (1.1 to 10.1)	<0.001	8.8 ± 1.9 (5.1 to 12.8)	9.2 ± 1.9 (5.9 to 13.3)	<0.001
Transverse diameter (cm)	25.9 ± 2.2 (21.1 to 32.0)	26.1 ± 2.2 (21.4 to 31.9)	0.181	24.7 ± 2.0 (21.5 to 29.2)	25.0 ± 2.0 (21.6 to 30.1)	0.004
Depth of depression (cm)	3.4 ± 1.3 (1.6 to 6.7)	3.4 ± 1.6 (0.7 to 7.5)	0.711	1.0 ± 0.5 (0.0 to 2.5)	0.9 ± 0.4 (0.0 to 1.9)	0.009
Vertebrae to thoracic cavity distance (cm)	6.0 ± 0.6 (4.3 to 7.4)	5.6 ± 0.5 (4.4 to 6.7)	<0.001	6.5 ± 0.6 (4.8 to 7.3)	5.6 ± 0.5 (4.7 to 6.8)	<0.001
Distance between T6 and T12 plumb lines (cm)	2.4 ± 1.0 (0.2 to 4.0)	0.8 ± 0.7 (−0.5 to 2.2)	<0.001	2.7 ± 1.1 (0.0 to 4.8)	1.5 ± 1.1 (0.0 to 6.0)	<0.001

Values are expressed as mean±SD (range).

**Figure 3:**
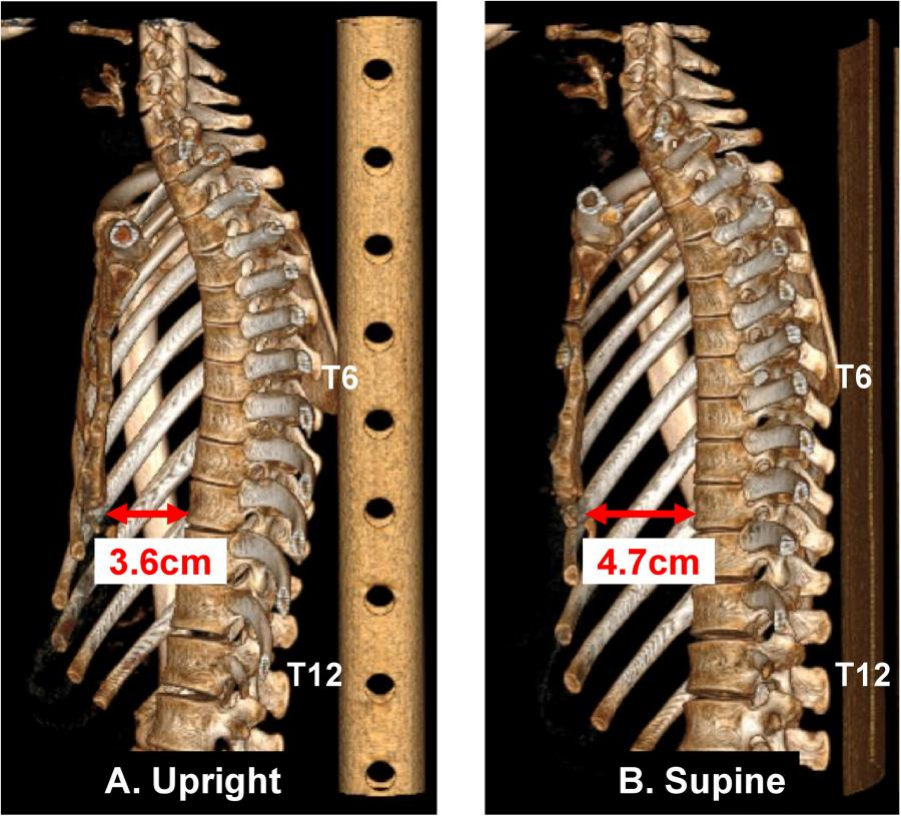
Representative sagittal computed tomography images in the upright (**A**) and supine (**B**) positions. In this patient, switching from the supine to the upright position resulted in a 1.9-cm anterior shift of the T12 plumb line relative to the T6 plumb line. The minimum anterior–posterior diameter (arrow) was shorter in the upright position than in the supine position (3.6 vs 4.7 cm).

The depth of depression (Fig. 2A, c) did not differ significantly between the 2 positions. In contrast, the vertebrae–thoracic cavity distance (Fig. 2A, d) was significantly longer in the upright position than in the supine position (6.0 ± 0.6 vs 5.6 ± 0.5 cm, *P* < 0.001). The distance between the plumb lines along the most anterior points of the T6 and T12 vertebral bodies on sagittal images (Fig. 2B) was significantly longer in the upright position than in the supine position (2.4 ± 1.0 vs 0.8 ± 0.7 cm, *P* < 0.001). This finding indicates that the T12 had shifted anteriorly while the degree of spinal curvature had increased in the upright position, as shown in the 3-dimenaional-reconstructed sagittal images of the spinal columns in a representative case (Fig. 3).

Healthy volunteers demonstrated a smaller minimum anterior–posterior diameter (8.8 ± 1.9 vs 9.2 ± 1.9 cm, *P* < 0.001), a greater HI (2.9 ± 0.6 vs 2.8 ± 0.5, *P* = 0.001), a longer vertebrae–thoracic cavity distance (6.5 ± 0.6 vs 5.6 ± 0.5 cm, *P* < 0.001), and a longer distance between the T6 and T12 plumb lines (2.7 ± 1.1 vs 1.5 ± 1.1 cm, *P* < 0.001) in the upright position than in the supine position (Table 3). The depth of depression differed significantly between the 2 positions (*P* = 0.009), although the difference in mean length was only 0.1 cm.

**Figure 4:**
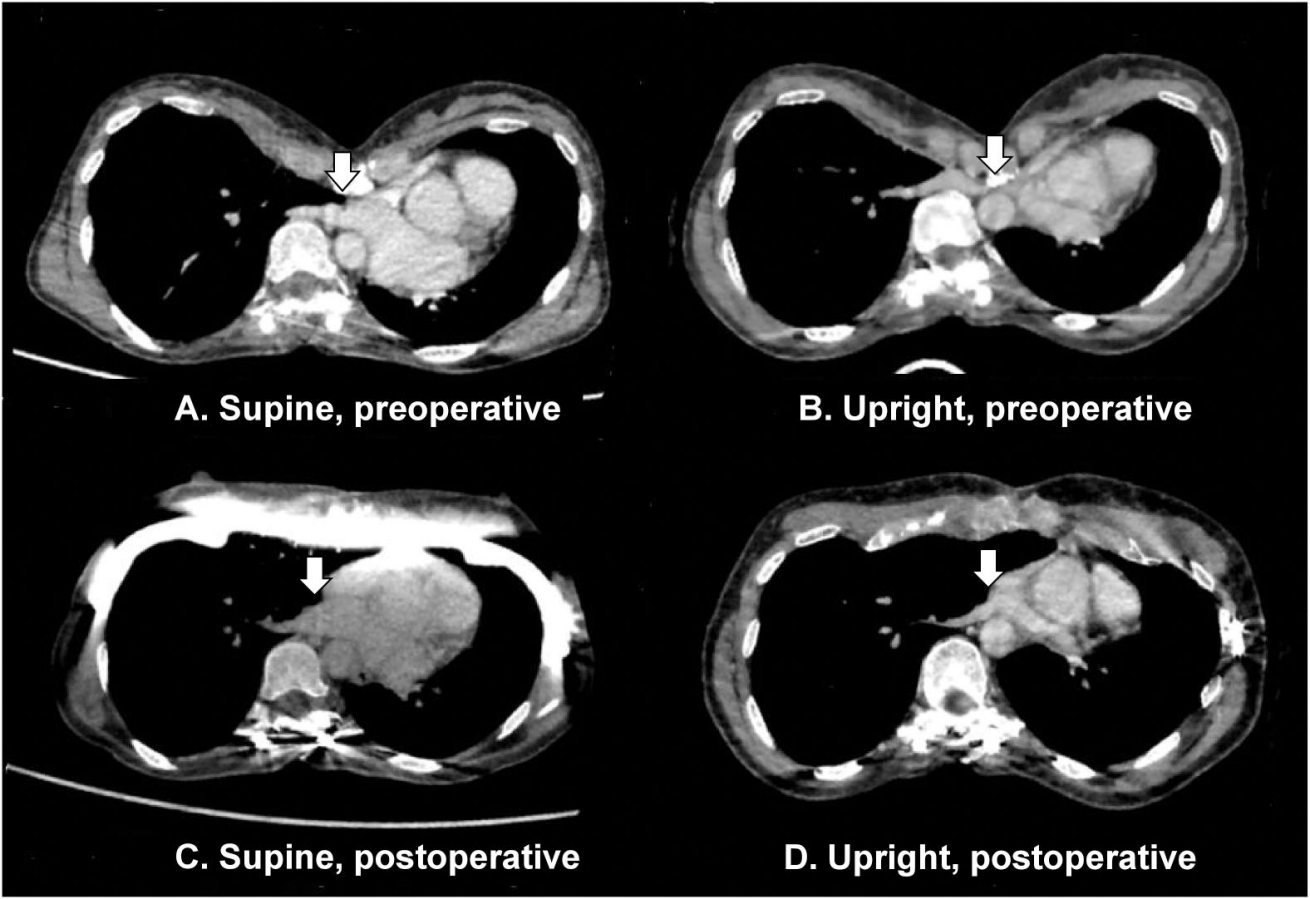
Horizontal computed tomography sections at the level of maximum chest depression from a 49-year-old patient with pectus excavatum presenting with dyspnoea, which worsened in the upright position. Images were captured during deep inspiration in the (**A**) supine position, preoperative; (**B**) upright position, preoperative; (**C**) supine position, postoperative; and (**D**) upright position, postoperative. The right inferior pulmonary vein (arrow) is significantly compressed in the upright position preoperatively (**B**).

In one case in our cohort, a 49-year-old female with dyspnoea that worsened in the upright position (Table 1), the minimum anterior–posterior diameter was significantly shorter in the upright position (0.3 vs 1.1 cm). Additionally, the right inferior pulmonary vein was compressed between the sternum and vertebra only on upright CT at the level of maximum chest depression (Fig. 4). After undergoing the Nuss procedure, her HI decreased to 5.4 in the upright position and 4.8 in the supine position, and her dyspnoea in the upright position improved.

## DISCUSSION

In this study, we used a newly developed upright CT method in addition to conventional supine CT to evaluate posture-related morphological changes in the chest wall, spinal column, and thoracic organs among 21 patients with PE and 35 healthy volunteers. In patients with PE, our examination showed that the HI was significantly greater and the minimum anterior–posterior diameter (an element of the HI) was significantly shorter in the upright position than in the supine position. While there was no significant difference in the depth of depression, the vertebrae–thoracic cavity distance was significantly longer in the upright than in the supine position. On sagittal images, the T12 plumb line shifted anteriorly from the T6 plumb line in the upright position compared to that in the supine position, indicating that the spinal column curved more in the upright position.

Notably, these posture-related changes in each measurement were also significant in healthy volunteers. This phenomenon could be consistent with human anatomy, irrespective of the presence of PE or other characteristics, including age, sex and body mass index (Table 1), which differed between patients with PE and volunteers in this study. Janssen *et al.* [18] previously investigated 30 asymptomatic volunteers using magnetic resonance imaging and reported that the curvature of the spinal column was greater in the upright position than in the supine position. Our results indicate that the lower thoracic vertebrae shift anteriorly in the upright position compared to that in the supine position, resulting in a shorter minimum anterior–posterior diameter and a greater HI in the upright position.

In both patients with PE and healthy volunteers (Table 3), the vertebrae–thoracic cavity distance significantly increased from the supine to the upright position. Although the change in the HI was significant in both groups, it was more pronounced in patients with PE (HI change in patients with PE: 6.4–10.1, HI change in healthy volunteers: 2.8–2.9). This may be primarily because the minimum anterior–posterior diameter was significantly shorter in patients with PE compared to that in healthy volunteers (measured by supine CT: 9.2 cm vs 5.1 cm, *P* < 0.001; Table 2). This indicates that an average anterior shift of the vertebrae by <1.0 cm resulted in a greater proportional shortening of the minimum anterior–posterior diameter in patients with PE.

The current results indicate that anterior shifting of the lower thoracic vertebrae in the upright position may worsen cardiovascular compression in patients with severe PE presenting with symptoms such as dyspnoea. Some previous studies on PE have also reported worsening of cardiac function and cardiopulmonary symptoms in the upright position [6, 10, 11, 19]. For example, Beiser *et al.* [11] performed catheterization of the right heart in 6 patients with PE. In their study, cardiac output during upright exercise was ‘low normal’ or lower in 5 patients, while all candidates demonstrated normal cardiac output in the supine position. Zhao *et al.* [19] examined exercise-related Doppler stroke volume during sitting and supine incremental cycling in 13 patients with PE, and the stroke volume was reduced in the sitting position compared to that in the supine position. These reports revealed that some patients with PE exhibit significantly decreased cardiac function in the upright position, which may explain the underlying mechanism.

Beiser *et al.* [11] previously indicated that the upright posture interferes with the cardiac pumping capacity by causing the heart to descend into the portion of the thorax narrowed by the depression of the lower sternum. Additionally, Zhao *et al.* [19] indicated that upright exercise capacity is impaired in patients with PE due to reduced heart filling in the upright position. However, we hypothesized that the posture-related changes in cardiac compression were caused by the anterior shift of the lower thoracic vertebrae, resulting in a shortened minimum anterior–posterior diameter of the thorax in the upright position. Furthermore, conventional supine CT may underestimate cardiovascular compression in patients with severe PE. Upright CT, or echocardiography in the upright position in centres where upright CT is unavailable, may be useful for evaluating the true impact of severe PE on circulatory dynamics.

This study has some limitations. First, because of the scheduling conflicts between preoperative participants and CT scanning, as well as a decrease in operative patients during the COVID-19 pandemic, we could evaluate only a relatively small number of patients. Additionally, although the preceding study (no. N20160384) initially enrolled 100 healthy volunteers, only 35 were scanned with arms down. Nonetheless, the shortening of the minimum anterior–posterior diameter and the subsequent increase in the HI were observed in 18 patients (86%), supporting the accuracy of our findings. Second, the method used to evaluate the anterior shift of the lower thoracic vertebrae based on the distance between the T6 and T12 plumb lines on sagittal CT was an original technique in this study. As the sagittal distance between the plumb lines of 2 individual vertebrae, such as the sagittal vertical axis, is widely accepted for the evaluation of spinal alignment, our method should be justifiable [20–22]. Third, we did not directly evaluate haemodynamic impairment in patients with severe PE, although we did observe mild posture-related radiological changes. Further investigations, including a combination of echocardiography and CT in both the upright and supine positions, are needed.

In summary, the minimum anterior–posterior diameter of the thorax decreases as the spinal column curvature increases, while the lower thoracic vertebrae shift anteriorly from the supine to the upright position. This phenomenon connects previously reported posture-related anatomical findings [18] and changes in circulatory dynamics observed in patients with PE [6, 10, 11, 18]. It may be the mechanism underlying the worsening of cardiopulmonary symptoms from the supine to the upright position in patients with severe PE. Upright CT could be instrumental in evaluating the true impact of severe PE on cardiovascular compression, which conventional supine CT may underestimate.

## Data Availability

The data underlying this article will be shared upon reasonable request to the corresponding author.

## Notes

Presented at the 23rd Annual Congress of the Chest Wall International Group in Istanbul, Turkey, 23rd June 2023, where it received the Young Investigator Award.

## ABBREVIATIONS

CT: Computed tomography
HI: Haller index
PE: Pectus excavatum
